# Oral oxymatrine for hepatitis B cirrhosis: A systematic review protocol

**DOI:** 10.1097/MD.0000000000013482

**Published:** 2018-12-10

**Authors:** Xiaotao Jiang, Linling Xie, Cihui Huang, Yishen Liu, Haining Liu, Binqian Liu, Liang Zheng

**Affiliations:** a Guangzhou University of Chinese Medicine; b Acupuncture and massage department, The First Affiliated Hospital of Guangzhou University of Chinese Medicine, Guangzhou, China.

**Keywords:** hepatitis B cirrhosis, oxymatrine, protocol, systematic review

## Abstract

**Background:**

Characterized by diffuse hepatic fibrosis and nodule formation, hepatitis B cirrhosis (HBC), an important result of chronic hepatitis B development, mainly contains compensated and decompensated stage. Compensated cirrhosis can further develop into decompensated stage and hepatocellular carcinoma with serious complications and high mortality. Antiviral therapy using interferon (IFN) or nucleos(t)ide analogs (NUCs) is essential for improving the prognosis of the disease but IFN has large side effects while NUCs often develop drug resistance. Antifibrosis is also an important strategy, but currently there is no effective antifibrosis drug. Pharmacologic studies have demonstrated that oxymatrine (OM) exhibits anti-hepatitis B virus (HBV) and antifibrosis effects. An increasing number of clinical controlled studies also have found that OM combined with conventional therapy could improve the curative effect and reduce adverse events incidence in treating HBC but there is no systematic review of it. Based on the extensive collection of literature, we will use meta-analysis to assess the efficacy and safety of OM for HBC.

**Methods:**

PubMed, MEDLINE, Embase, Cochrane Library, China National Knowledge Infrastructure, Wanfang data, Chinese Scientific Journals Database (VIP), and China biomedical literature database will be searched to obtain the eligible studies published up to July 15, 2018. The primary outcome will be liver function indexes, liver fibrosis indexes, and Child–Pugh score. The secondary outcome will be hepatitis B virus DNA quantification, HBV DNA seroconversion rate, hepatitis B e antigen (HBeAg) seroconversion rate, and adverse events incidence. Data analysis will be conducted using RevMan 5.3 and Stata V.9.0 software. Trial sequential analysis (TSA) will be performed to assess the risk of random error and the validity of conclusion using TSA program version 0.9 beta.

**Results:**

This systematic review will provide a high quality synthesis of OM for HBC from various evaluation aspects including liver function indexes, liver fibrosis indexes and Child-Pugh score, HBV DNA quantification, HBV DNA seroconversion rate, HBeAg seroconversion rate and adverse events incidence.

**Conclusion:**

The systematic review will provide evidence to assess the efficacy and safety of OM in the treatment of HBC.

**PROSPERO registration number:**

PROSPERO CRD42018095275.

## 1 Introduction

The latest clinical practice guidelines published by European Association for the Study of Liver (EASL) point out that about one-third of the world's population has been or is infected with hepatitis B virus (HBV), and over 0.5 to 1 million people die of HBV-related end-stage liver diseases or hepatocellular carcinoma (HCC) per year.^[1]^ It is reported that approximately 20% of patients with chronic hepatitis B (CHB) develop hepatitis B cirrhosis (HBC) and annually about 3% patients with CHB develop cirrhosis.^[2,3]^ Characterized by diffuse hepatic fibrosis and nodule formation, HBC, an important result of CHB development, mainly contains compensated and decompensated stage.^[1,4]^ Compensated cirrhosis can further develop into decompensated stage and HCC with serious complications and high mortality.^[5]^ The 5-year survival rates of patients with compensated and decompensated cirrhosis were 84% and 14%, respectively.^[6]^ Therefore, it is important to postpone or block the development of HBC.^[7]^ Antiviral therapy is essential for improving the prognosis of the disease, and it is also an important part of the overall treatment strategy of HBC.^[8]^ Studies have demonstrated that effective inhibition of HBV replication can improve hepatic fibrosis,^[8–10]^ delay or prevent the cirrhosis progression from compensated to decompensated stage. Besides, effective inhibition of HBV replication can reduce further deterioration of patients in decompensated stage and occurrence of portal hypertension as well as related complications,^[11–14]^ thus extending survival time. Interferon (IFN) and nucleos(t)ide analogs (NUCs) including lamivudine (LAM), adefovir (ADV), entecavir (ETV), telbivudine (LDT), and tenofovir (TLV) are the commonly used antiviral drug clinically. However, IFN with large side effects can cause liver failure and is forbibden to use in decompensated cirrhosis.^[1]^ NUCs with less adverse reactions but often develop drug resistance.^[15]^ Antifibrosis is also an important strategy, but currently there is no effective antifibrosis drug.

Oxymatrine (OM), isolated from the Sophora alopecuroides L and radix Saphora flavescens Ait, exhibits potent effects on antiinflammation, antivirus, immune regulation, and antifibrosis.^[16]^ It has been demonstrated that OM exerts direct anti-HBV effect in vitro^[17]^ and can reduces content of hepatitis B surface antigen (HBsAg) as well as hepatitis B e antigen (HBeAg) in vivo.^[18]^ It also showed an antifibrosis effect with suppressing the proliferation of hepatic stellate cell as well as the mRNA expression of procollagen type III (PC-III).^[19]^ Further study revealed that OM is able to promote the degradation of extracellular matrix by inhibiting the secretion of transforming growth factor-β1 and transforming growth factor-α1 from interstitial cells and hepatocytes which are considered as the key factors of liver fibrosis formation, thus reducing the levels of hyaluronic acid (HA), PC-III, type IV collagen (IV-C), laminin (LN) in serum, and exerting the functions of antihepatic fibrosis.^[20]^ For its anti-HBV and antifibrosis effects, some physicians in China use it in the treatment of HBC. An increasing number of clinical controlled studies also have found that OM combined with conventional therapy could improve the curative effect and reduce adverse effect in treating HBC. However, there is no systematic review of it. Based on the extensive collection of literature, we will use meta-analysis to assess the efficacy and safety of OM in the treatment of HBC.

## 2 Methods

### 2.1 Study registration

The protocol has been registered in the International Prospective Register of Systematic Reviews (PROSPERO) with registration number CRD42018095275 on June 11, 2018.

### 2.2 Ethics and dissemination

Ethical approval is not required as there are no issues about participants’ privacy in our research. We aim to publish the results in a peer-reviewed journal. The results of this review will provide information about the safety and efficacy of OM taking orally in the treatment of HBC and help clinicians make decisions on clinical practice.

### 2.3 Types of studies

All randomized controlled trials (RCTs) comparing the effect and safety of OM taking orally on HBC will be included, regardless of the stage of cirrhosis and the length of treatment.

### 2.4 Participants

Patients who have been diagnosed with HBC based on the Viral Hepatitis Prevention and Treatment Programs Revised in Xi’an Conference^[21]^ or 2010 Chinese Guidelines for the Management of Chronic Hepatitis B^[22]^ will be included, regardless of compensated or decompensated stage, age, and sex. Patients with definite diagnosis of cirrhosis should be based on liver biopsy or laboratory, imaging examination compatible with the diagnosis of HBC. Patients with CHB but not developing into cirrhosis, patients with liver cirrhosis unrelated to HBV infection, patients with liver cirrhosis with combined HBV and HCV infection, and patients with HCC will be excluded.

### 2.5 Types of interventions

In experiment group, oral OM, regardless of dosage, will be used combined with conventional treatments such as LAM, ETV, ADV, LDT, IFN, and etc. Patients of control group will be treated with conventional therapy in accordance with experiment group.

### 2.6 Outcome measures

#### 2.6.1 Primary outcomes

1. Liver function indexes: alanine aminotransferase (ALT), serum total bilirubin (TBIL), aspartate aminotransferase (AST), albumin (ALB)
2. Liver fibrosis indexes: LN, HA, PC-III, IV-C
3. Child–Pugh score

#### 2.6.2 Secondary outcomes

1. HBV DNA quantification
2. HBV DNA seroconversion rate
3. HBeAg seroconversion rate
4. Adverse events incidence

### 2.7 Search strategy

PubMed, MEDLINE, Embase, Cochrane Library, China National Knowledge Infrastructure (CNKI), Wanfang data, Chinese Scientific Journals Database (VIP), and China biomedical literature database will be search and eligible studies published up to July 15, 2018 will be acquired. Various combinations of Medical Subject Headings and non-MeSH terms will be used, including “oxymatrine,” “kurorinone,” “kushensu,” “kushenin,” “cirrhosis,” “liver cirrhosis,” “hepatitis B,” and “HBV,” which will be searched individually or in combination. Language, population, or country restrictions will not be applied.

The specific search strategy will be (taking PubMed as an example):

1. Oxymatrine[MeSH] OR kurorinone[Title/Abstract] OR kushensu[Title/Abstract] OR kurorinone[Title/Abstract] OR kushenin[Title/Abstract]
2. Cirrhosis[MeSH] OR liver cirrhosis[Title/Abstract] OR hepatitis B[Title/Abstract] OR HBV[Title/Abstract]
3. 1 AND 2
  The strategy will be modified for other databases use if necessary. The reference lists of the relevant articles will also be checked whether they are eligible to be pooled.

### 2.8 Data collection and analysis

#### 2.8.1 Study selection

**Figure 1:**
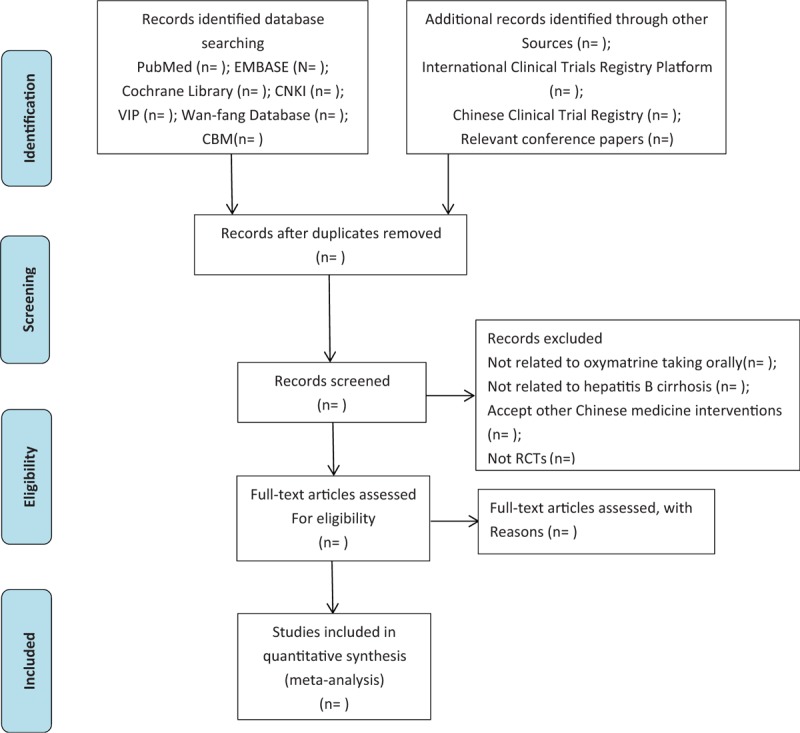
Flow diagram of study selection process.

The RCTs comparing the effect and safety of OM on HBC will be selected. Those articles meeting one of following items will be excluded: the duplicates, the participants did not meet the diagnosis criteria of HBC or the diagnosis criteria is unknown, not RCT studies, the studies in which the experimental participants do not receive OM taking orally in combination with conventional therapy as the primary intervention, the intervention contains any other traditional Chinese medicine therapy, and incomplete data which will be needed. Two reviewers will assess whether the studies are eligible. Group discussion will be required when there is any disagreement during the articles inclusion. The specific process of studies screening will be displayed in a Preferred Reporting Items for Systematic Reviews and Meta-Analyses (PRISMA) flow diagram (Fig. 1).

#### 2.8.2 Data extraction and management

Data will be independently extracted from the pooled studies by 2 reviewers, with all reviewers discussing any disagreements raised during the data extraction process. The following information will be extracted: study details (authors, country, year of publication, multicenter or single center study), participant details (baseline data, diagnostic criteria, HBC stage), the methods used (registry platform, sample size, blinding method), the interventions and the outcomes (liver function indexes, liver fibrosis indexes, Child–Pugh score, HBV DNA quantification, HBV DNA seroconversion rate, HBeAg seroconversion rate, adverse events incidence). We will contact the corresponding author for the data mentioned above if they are not reported. All reviewers will discuss to solve the disagreement.

### 2.9 Risk of bias assessment

Two authors will independently evaluate the risk of bias of each included article on the basis of the Cochrane Handbook for Systematic Reviews of Interventions. The methodologic quality will be appraised from the following seven aspects: random sequence generation, allocation concealment, blinding of participants and personnel, blinding of outcome assessments, incomplete outcome data, selective reporting, and other bias. Each aspect above of each pooled study will be described to provide the rationale for the risk judgment. The risks will be categorized as low, high or unclear displaying in graphical form.

### 2.10 Data synthesis

We will use RevMan 5.3 software to analyze the related research indicators. For dichotomous outcomes, HBV DNA seroconversion rate and HBeAg seroconversion rate will be expressed by risk ratios with 95% confidence intervals (CIs), while adverse events incidence expressed by odds ratio with 95% CIs. Continuous data including liver function indexes, liver fibrosis indexes, Child–Pugh score, and HBV DNA quantification will be expressed by the weighted mean difference with 95% CIs.

### 2.11 Dealing with missing data

We will attempt to contact the corresponding author through email if there exists any missing information which is required. If missing data cannot be obtained, we will analyze the available data and discuss what it might cause for the review.

### 2.12 Assessment of heterogeneity

Heterogeneity among the studies will be evaluated using the *I*^2^ statistic. *I*^2^ > 50% indicates a significant heterogeneity, and under this circumstance, subgroup analysis, influence analysis, as well as meta-regression analysis will be performed to ascertain the potential causes from clinical or methodologic heterogeneity. The choice of random effect model or fixed effect model will depend on whether there exists heterogeneity or not. If heterogeneity exists but cannot be explained reasonably and consequently meta-analysis is unavailable to be conducted, we will describe the data.

### 2.13 Subgroup analysis

Subgroup analysis based on cirrhosis stage, treatment period, dose of OM, level of risk of bias, and other unpredictable factors will be performed to detect the source of heterogeneity.

### 2.14 Sensitivity analysis

Sensitivity analysis will be performed to evaluate the methodologic and reporting quality of the pooled studies.

### 2.15 Assessment of reporting biases

If the amount of pooled studies is ≥10, Egger or Begg test will be conduct to analyze the potential publication bias using Stata V.9.0. If publication bias exists, shearing and mending method will be conducted to assess the impact of publication bias on the results.

### 2.16 Trial sequential analysis

To solve the problem about the repeated significance test on cumulative data increasing the overall risk of I errors in a single RCT, statistical monitoring boundaries will be adopted to assess if a single test could reach an in-advance termination with a *P*-value small enough to show the expected effect or futility.^[23,24]^ Trial sequential analysis (TSA) is always applied in the risk of type I errors assessment by combining information size estimation with an adjusted threshold for statistical significance in the cumulative meta-analysis.^[23,24]^ Trial sequential monitoring boundaries calculated using Obrien–Fleming alpha spending function adjusts the CIs and reduce type I errors. The reliability of expected intervention efficacy would be evidenced, if the trial sequential monitoring boundaries are crossed by the cumulative *z* curve, and under this circumstance, no further trials should be conducted.^[25]^ If cumulative *z* curve does not go through the boundaries and required information size, an stable conclusion cannot be drawn.^[26]^ In our assumption, type I error will be α = 0.05 and type II error will be set at β = 0.20. Relative risk reduction and the incidence in control arm will be derived from the meta-analysis outcomes. TSA 0.9 (Copenhagen Trial Unit, Copenhagen, Denmark) will be used to perform TSA to assess the risk of random error and the validity of conclusion.

### 2.17 Quality of evidence

We will adopt The Grading of Recommendations Assessment, Development, and Evaluation approach to assess the quality of evidence of the pooled studies. Limitations of the study, inconsistencies, indirect evidence, inaccuracies, and publication bias will be considered. Levels of evidence quality will be classified into four levels: high, moderate, low, or very low.

## 3 Discussion

It is reported that, for patients with CHB without treatment, the 5-year cumulative incidence of developing cirrhosis ranges from 8% to 20%. For those untreated patients with compensated cirrhosis, the 5-year cumulative incidence of hepatic decompensation is approximately 20%. The 5-year survival rate of decompensated cirrhosis patients without treatment is 14% to 35%.^[27–32]^ Therefore, effective and safe management of the cirrhosis stage is necessary. Antiviral therapy and antifibrosis are essential to postpone or block the development of HBC.^[7]^ However, drug resistance often occurs as HBV often mutates, therefore avoiding the effect of NUCs which is a type of antiviral drugs commonly used at present. Besides, effective antifibrosis drugs are not available up to now. OM have been demonstrated antiviral and antifibrosis effects from pharmacologic perspective. In clinical controlled studies, it also shows the ability to enhance efficacy and reduce side effects in HBC treatment. To our knowledge, there is no related systematic review evaluating the impact of OM for HBC. Therefore, we will conduct this systematic review to further evaluate the effectiveness and safety of OM for HBC. Our aim is to provide more clinical evidence helping clinicians make decisions on clinical practice in HBC treatment.

## Author contributions

Xiaotao Jiang, Linling Xie and Liang Zheng designed the study. Xiaotao Jiang and Linling Xie drafted the protocol. All authors revised the manuscript. All authors approved the final version.

Conceptualization: Xiaotao Jiang, Linling Xie, Cihui Huang.

Methodology: Xiaotao Jiang, Liang Zheng.

Writing-original draft: Xiaotao Jiang, Linling Xie.

Writing-review & editing: Xiaotao Jiang, Linling Xie, Cihui Huang, Yishen Liu, Haining Liu, and Binqian Liu.

Supervision: Liang Zheng.

**Conceptualization:** Xiaotao Jiang, Linling Xie, Cihui Huang.

**Methodology:** Xiaotao Jiang.

**Supervision:** Liang Zheng.

**Writing – original draft:** Xiaotao Jiang, Linling Xie.

**Writing – review & editing:** Xiaotao Jiang, Linling Xie, Cihui Huang, Yishen Liu, Haining Liu, Binqian Liu.
